# Protective Effect of Red Okra (*Abelmoschus esculentus* (L.) Moench) Pods against Sodium Nitrite-Induced Liver Injury in Mice

**DOI:** 10.1155/2021/6647800

**Published:** 2021-06-16

**Authors:** Sri Puji Astuti Wahyuningsih, Adamu Ayubu Mwendolwa, Dwi Winarni, Rizki Wahyu Anggreini, Brigita Klara Krisdina Mamuaya

**Affiliations:** ^1^Department of Biology, Faculty of Science and Technology, Airlangga University, Surabaya 60115, Indonesia; ^2^Department of Biological Sciences, Faculty of Sciences, Mkwawa University College of Education, P.O. Box 2513, Iringa, Tanzania

## Abstract

Vegetables, drinking water, and preserved meats may contain sodium nitrite (NaNO_2_), which causes liver disease by inducing oxidative stress. Phytochemicals are highly recommended as an alternative to synthetic drugs and affordable medicines to treat liver disease because they have fewer or no side effects. Therefore, this study aims to determine the antioxidant and hepatoprotective potential of red okra fruit ethanol extract against NaNO_2_-induced liver damage. Thirty-six male mice were separated into six groups. The normal control group (WA) was given distilled water only, and the NaNO_2_ (SN) group was given only 50 mg/kg BW NaNO_2_. The other four groups (P1, P2, P3, and P4) were given NaNO_2_ and red okra ethanol extract at doses of 25, 50, 75, and 100 mg/kg BW, respectively. Gavage was administered orally for 21 consecutive days. Commercial kits define all biochemical parameters according to the manufacturer's instructions. Liver tissue staining followed standard protocols using hematoxylin and eosin. The study revealed that NaNO_2_ induction causes oxidative stress and damages the liver. The activity of antioxidant enzymes (superoxide dismutase and catalase) significantly increased in the groups treated (P2–P4) with ethanol extract of red okra (*p* < 0.05). Besides, the oxidants (malondialdehyde, F2-isoprostanes, and nitric oxide) in the liver homogenate significantly decreased in the P4 group, which were given red okra ethanol extract (*p* < 0.05). Likewise, red okra pods decreased significantly for the serum biochemical parameters of liver damage (aspartate aminotransferase, alkaline phosphatase, and alanine aminotransferase) in the P3 and P4 groups (*p* < 0.05). Then, it led to a restoration of the histological structure compared to exposed mice (SN), as the pathological scores decreased significantly in the P3 and P4 groups (*p* < 0.05), as well as the number of the necrotic and swollen liver cells was reduced. Hepatocytes returned to normal. The results showed that the ethanol extract of red okra fruit could be helpful as an affordable medicine. It is an antioxidant and hepatoprotective agent to protect the liver from damage caused by NaNO_2_.

## 1. Introduction

Hepatic diseases are global deadly health problems that cause human deaths. It is estimated that around 2 million deaths each year in the world is associated with liver diseases. Acute hepatitis, cirrhosis, liver cancer, and alcohol-associated liver disease are more common, causing 4% of all deaths worldwide; the percentage is expected to rise due to lifestyles with little or no physical activities and over nutrition [1, 2]. Intrinsic and extrinsic factors such as being born with metabolic defects, malnutrition, viral infection, and exposure to toxic substances [3] may produce reactive nitrogen species/reactive oxygen species (RNS/ROS), facilitating liver disease illness. The liver is a vital organ responsible for the detoxification of poisonous materials such as xenobiotics. It plays various roles in metabolism, secretion of wastes, and elimination of unneeded materials from the body; hence, it is the main target of toxins and xenobiotics [4, 5]. Natural and artificial manufactured food additives increase and maintain food safety, texture, taste, and nutrient value. Although they are beneficial, synthesized additives may cause some adverse effects [6]. One of the synthesized food additives is sodium nitrite (NaNO_2_), which can be present in vegetables, cured meat, and fish. Human activities such as agriculture, which involves using nitrogenous fertilizer to increase crop productions, improper handling, and maintenance of industrial and sewage waste, increase the risk of NaNO_2_ exposure to humans. Around the world, drinking water has been occasionally found to contain a level of NaNO_2_ beyond the acceptable limit [7]. Nitrite has various functions, ranging from physiological, immunological to neurological functions at a low physiological amount. In the body, NaNO_2_ can be interconverted into various kinds of nitrogen molecules, including nitric oxide (NO) that improves digestive health by facilitating gastric flow, preserving the integrity of the gastric epithelium and muscular barrier [8], inhibiting white blood cells from adherence to the endothelium as well as decreasing the risk of cardiovascular diseases and improving the pulmonary health. In a clinical setting, NaNO_2_ is used as an antidote for cyanide poisoning [9]. In food industries, NaNO_2_ is used in meat and fish as a color fixative agent to improve flavor, delay rancidity by preventing fat oxidation, and inhibit the growth of microorganisms such as *Clostridium perfringens*, *Staphylococcus aureus*, and *Bacillus cereus,* as well as *C. botulinum* that causes botulism [8, 10, 11].

Despite the health benefits of NaNO_2_ at low concentrations, thanks to the NO that can be produced from it, howbeit the compound is detrimental at high concentrations because of its ability to oxidize various molecules such as protein, lipid, and deoxyribose nucleic acid (DNA) [5, 8]. Exposure to NaNO_2_ causes methemoglobinemia, hypoxia, and the formation of carcinogenic nitroso compounds due to their interaction with amide and amine in the stomach at low pH [8, 12]. The nitroso compounds and other forms of nitrite generate free radicals such as superoxide (O_2_), peroxynitrite (ONOO), and hydroxyl radicals (OH) that cause cellular damage by lipid peroxidation, protein oxidation, carbohydrate carboxylation, and DNA damage leading to cancer, congenital disabilities, dysregulation of inflammatory responses, tissue injuries, nephrotoxicity, impairment of the reproductive system, disturbance of the endocrine system, growth retardation, and hepatotoxicity [5, 6, 13].

Several approaches have been used for a long time to treat liver disorders, such as vaccines, corticosteroids drugs [14], and liver transplantation [4]. The approaches have some limitations, including serious side effects when used for a long time, and have limited ability to produce the desired impacts [4, 14]. Alternatively, the use of natural products such as compounds from plants and their derivatives have shown promising results because they are less toxic and have little or no side effects [4, 14, 15].

Okra (*Abelmoschus esculentus* L.) Moench is a flowering plant in the Malvaceae family. It is also named ladies' finger, bamyah, bamieh, kacang, gumbo, dharos, bhindi, bendi [16], or bamia in a particular region of the world. Various parts of the red okra plant have many benefits to the body. Fresh okra heals constipation, leucorrhea, spermatorrhea, diabetes, and jaundice; the mucilage can cure diarrhea, dysentery, gastric ulcer [14, 17], and syphilis [3], and when mucilage of okra is mixed with a ripe banana can be used to treat colitis, cystitis, hepatitis, and jaundice. The polysaccharides from okra modulate and improve organisms' immune response due to *S. aureus* infection [15]. Okra pods contain polyphenols and flavonoids such as quercetin that have higher antioxidant activity [18–20] can scavenge free radicals and decrease oxidative stress in the cells. The polyphenols and flavonoids can also protect the liver from the toxic effects of xenobiotics intoxication [3, 14]. Specifically, red okra pods have added xenobioticsidants (anthocyanin) responsible for the pods' red color [21]. Extracts from purple okra contain anthocyanin with higher antioxidant and quercetin content than the green okra; hence, plants containing anthocyanin are more effective than the plant devoid of the compound [22]. Okra is a popular vegetable crop with good nutritional significance and specific therapeutic values, making it a potential candidate for the use of a variety of nutraceuticals and affordable medicines. The antioxidant and hepatoprotective activity of red okra pods on liver injury induced by sodium nitrite has not been reported to the best of our knowledge. Therefore, this study was done to investigate the antioxidant and hepatoprotective activity of ethanol extract of red okra pods against sodium nitrite-induced liver injury in mice (*Mus musculus*).

## 2. Materials and Methods

### 2.1. Chemicals and Reagents

Ethanol, *n*-hexane, and ethyl acetate were purchased from Fulltime Chemical (Anhui, China). Superoxide dismutase- (SOD-) Typed Assay kits (E. BC-K022-5) and Elabscience®8-epi-PGF2*α* (8-Epi-Prostaglandin F2 Alpha) Elisa kits were purchased from Elabscience Biotechnology Inc. (Hayward, USA). In contrast, CAT kits (EC 1.11.1.6, K#K773-100) were purchased from BioVison Inc. (California, USA). Aspartate aminotransferase (ASAT) FS^*∗*^(IFCC mod) 12601991021, alkaline phosphatase (ALP) FS^*∗*^(IFCC mod) 104019910021, and alanine aminotransferase (ALAT) FS^*∗*^(IFCC mod) 127019910021 were purchased from Diasys Diagnostic system (Holzheim, Germany). Pierce™ Coomassie (Bradford) Protein Assay Kit (no. 23200) was purchased from Thermo Scientific (Rockford, USA); Bioxytech® MDA-586 spectrophotometric assay kit was purchased from Oxis International, Inc. (Portland, USA); phosphoric acid solution, sulfanilic acid, and *N*-(1-naphthyl)-ethylenediamine were purchased from Merck (Darmstadt, Germany). Neutral-buffer 10% formalin, Entellan, paraffin, alcohol hematoxylin, and eosin were purchased from a validated dealer in Surabaya, Indonesia. The study used chemical reagents that were of pure analytical grade.

### 2.2. Extraction of Plant Material

Red okra pods were purchased from a local market in Jember, Indonesia, identified and verified by Lembaga Ilmu Pengetahuan Indonesia, LIPI (Indonesia Institute of Sciences) with a certificate number 1204/IPH.06/HM/XI/2019. Plant materials were extracted by an exhaustive serial extraction using ethyl acetate, *n*-hexane, and ethanol. For the first two solvents, ethyl acetate, *n*-hexane extraction is done by the protocol of Wahyuningsih et al. [20], followed by ethanol extraction using the protocols of Yasin et al. [23]. Briefly, 5 kg of red okra pods was washed and cut into small pieces, dried without direct exposure to sunlight for seven days; after that, okra pieces were crushed with a shredder to produce a powder. The red okra powder was filtered by sieve no. 40 and stored in the desiccator. The phytochemicals were extracted using *n*-hexane three times in 24 hours, followed by ethyl acetate (three times, 24 hours each). The residues obtained were soaked in 500 mL of pure ethanol for 24 hours three times with constant stirring. Maceration results were filtered, and the filtrates were collected and then evaporated at 60°C in a rotary evaporator to get the ethanol extract. Finally, the extracts were freeze-dried to remove the solvents and then stored at −20°C for further use. The ethanol extract was used in animal treatment from the *n*-hexane, ethyl acetate, and ethanol extracts since Thavamoney et al. [24]. The ethanol extract had higher total flavonoid, phenolic, and anthocyanin content than the *n*-hexane and ethyl acetate extracts.

### 2.3. Animals and Experimental Design

Thirty-six adult male mice (*M. musculus*), BALB/c strain, aged 8–10 weeks, were purchased from Bratang Market, Surabaya, Indonesia, and acclimated for two weeks. Mice were housed in plastic cages (2 mice per cage) covered with wire gauze in a ventilated room with standard conditions with a 12-hour light-dark cycle in the animal laboratory, Faculty of Science Technology, Airlangga University, Indonesia. The mice were divided into six groups, each containing six mice. The groups were normal control (WA), which was given 0.2 mL of distilled water daily, NaNO_2_ group (SN), which was given 0.2 mL NaNO_2_ at a 50 mg/kg BW dose daily. Other groups were P1, P2, P3, and P4 that were given 0.2 mL at doses of 25, 50, 75, and 100 mg/kg BW of ethanol extract of red okra pods together with 0.2 ml of 50 mg/kg BW NaNO_2_ daily. Ethanol extract from red okra was given to the mice 30 min after the administration of NaNO_2_. All treatments were given by oral gavage. During the 21 days of the treatment, the mice had free access to food and water. The Animal Care and Use Committee (ACUC) of the Faculty of Veterinary Medicine, Universitas Airlangga, approved this study (2.KE.057.04.209).

### 2.4. Sample Collection

The mice were sacrificed by using ketamine 10% anesthesia. A disposable syringe collected blood from the left ventricle. The blood was left to stand in the microtube for two hours at room temperature, and after forming two phases, the liquid phase was collected. The sample obtained was stored at 4°C for further use [15]. The serum was used to determine the AST, ALT, and ALP activities, and TSP concentration.

The preparation of liver homogenates followed the procedures of Cheng [25] with few modifications. The liver was cut to form small pieces then crushed with a mortar and pestle in the mixture of 5 mL phosphate buffer saline (PBS). The suspension was filtered by using a 200 *μ*M mesh filter centrifuged at 1500 rpm for 5 min. The pellets obtained were resuspended in Tris-buffered NH_4_Cl, pH 7.2, to lyse the hepatocytes, centrifuged at 1500 rpm for 5 min. The procedure was repeated until white pellets were obtained. The pellets were sonicated at 20 kHz for 20 × 6 under ice-cold water. They were then centrifuged at 2500 rpm for 5 min to obtain the tissue homogenates stored at 4°C. The liver homogenates were used to determine SOD, CAT, MDA, NO, and F2-Isoprostanes levels.

### 2.5. Determination of SOD and Catalase from the Liver Homogenates

The SOD activity was determined by the superoxide dismutase- (SOD-) Typed Assay kits (E. BC-K022-5) as per the kit manufacturer's instructions. The absorbances were read at wavelength 550 nm using a spectrophotometer (Thermo Scientific™ Multiskan™GO), and the activity of SOD was calculated based on the manufacturer's instructions.

The CAT activity was determined by CAT (EC 1.11.1.6, K#K773-100) of BioVision as per the manufacturer's instructions. Shortly, 50 *μ*L of the liver homogenates was put into a well plate; then, 28 *μ*L of the assay buffer was added, followed by 12 *μ*L mMH_2_O_2_ to start the reaction. The plate was incubated at 25°C for 30 min; then, 10 *μ*L of stop solution was added, followed by 50 *μ*L of developer mix. After the incubation at 25°C for 10 min, the optical density (OD) values at wavelength 570 nm were read in a microplate reader (Thermo Scientific™ Multiskan™GO). CAT activity was calculated as instructed by the kit manufacturer.

### 2.6. Determination of MDA and NO

The MDA level was determined using Bioxytech® MDA-586 spectrophotometric assay kit, while NO was determined by phosphoric acid, sulfanilic acid, and *N*-(1-naphthyl)-ethylenediamine as reported by Wahyuningsih et al. [20]. MDA and NO absorbances were measured at wavelengths 586 nm and 540 nm, respectively, using a microplate UV-Vis spectrophotometer.

### 2.7. Determination of F2-Isoprostane

The concentration of F2-isoprostane in the liver was determined by Elabscience®8-epi-PGF2*α* (8-Epi-Prostaglandin F2 Alpha) Elisa kit as per the manufacturer's instructions. The absorbances were recorded at the wavelength of 450 nm with a microplate reader (Thermo Scientific™ Multiskan™GO).

### 2.8. Determination of Liver Enzymes

The activities of AST, ALP, and ALT were determined by ASAT (GOT) FS^*∗*^(IFCC mod) 12601991021, Alkaline Phosphatase FS^*∗*^(IFCC mod) 104019910021, and ALAT (GPT) FS^*∗*^(IFCC mod.) 127019910021 kits, respectively, in harmony with the manufacturer's directives. The OD values were read using a UV-Vis spectrophotometer at wavelength 365 nm for both AST and ALT, and 405 nm for ALP.

### 2.9. Determination of Concentration of TSP

The concentration of TSP was determined by Pierce^TM^ Coomassie (Bradford) Protein Assay Kit (no. 23200) as recommended by the kit manufacturer. The absorbances were read at wavelength 595 nm by a UV-Vis spectrophotometer.

### 2.10. Histological Examination of the Liver

The liver histological preparation and staining followed H&E staining's standard protocols, described by Feldman and Wolfe and Salahshoor et al. [26, 27]. In summary, the liver tissue was cut into two sections and fixed into buffer formalin 10% and then embedded into paraffin blocks. The liver sections in the paraffin blocks were sliced by microtome to form sections of approximately 3 *μ*m. The staining protocols for hematoxylin and eosin were followed, then the entellan mounting medium was added before covering the slide with a cover glass. Inflammation was scored following the method reported by Giribabu et al. [28] and El-Nabarawy et al. [12] based on the number of foci recognized under the low-power field of a light microscope (100X). The inflammation grades were 0 when there was no focus of inflammation, 1 when there was one focus per low-power field of inflammation, 2 when there were two foci per low-power field of inflammation, and 3 when there was 3+ foci low-power field of inflammation. Observations were made on an Olympus 1 × 51 light microscope at the magnification of 400X to count the necrotic, normal, and swollen hepatocytes. The use of ImageJ 1.53a did the counting of the cells. All photos were taken with an Olympus DP-10 digital camera.

### 2.11. Data Analysis

Data were statistically analyzed using the Statistical Package for the Social Sciences (SPSS) 26.00 for windows. The data were analyzed by a one-way analysis of variance (one-way ANOVA) followed by the Duncan or Games-Howell, and the Kruskal–Wallis followed by the Mann–Whitney test. The data were presented as mean ± standard error (M ± SE), and the difference of *p* < 0.05 was considered statistically significant.

## 3. Results

### 3.1. The Effect of Ethanol Extract from Red Okra Pods on SOD and CAT Activities

The ethanol extract of red okra pods on SOD and CAT activities is presented in Table 1. NaNO_2_ caused a significant decrease in the activity of SOD in the mice that were given NaNO_2_ alone (SN) compared to the mice given water alone (WA). The ethanol extract of red okra caused a significant increase in the activity of the SOD in all red okra-treated groups compared to SN. The activity of CAT was significantly decreased in SN compared to WA. CAT activity was significantly increased in mice given red okra extract except in the group that received 25 mg/kg BW.

### 3.2. The Effect of Ethanol Extract from Red Okra Pods on MDA and F2-Isoprostanes

The effects of the ethanol extract of red okra pods on MDA and F2-Isoprostanes levels in mice are presented in Table 1. NaNO_2_ caused a significant increase in MDA and F2-isoprostanes in SN's liver compared to WA. The administration of ethanol extract of red okra pods ameliorated MDA and F2-isoprostanes by decreasing their liver homogenates' concentrations.

### 3.3. The Effect of Ethanol Extract from Red Okra Pods on NO

The NO concentration caused a significant increase in the SN compared to WA. However, the administration of ethanol extract from red okra leads to a decrease in NO in all mice given red okra extract compared to SN.

### 3.4. The Effect of Ethanol Extract from Red Okra Pods on Liver Enzyme Activities

The results of the ethanol extract of red okra pods on the activity of the liver enzymes in the serum of mice exposed to 50 mg/kg BW are presented in Table 2. The administration of NaNO_2_ caused a significant increase in the ALT activity in the serum SN by two folds compared to WA. Compared to SN, the ethanol extract of red okra significantly decreased ALT activity in all groups given red okra extract except P1. Mice exposed to NaNO_2_ alone showed a significant increase in AST activities compared to WA. The activity was significantly decreased in the mice administered with 75 and 100 mg/kg BW red okra extract together with NaNO_2_.

### 3.5. The Effect of Ethanol Extract from Red Okra Pods on the Concentration of TSP

The ethanol extract of red okra pods on the TSP concentration in mice is presented in Table 2 NaNO_2_ caused a significant decrease in the concentration of TSP in the serum compared to WA. The red okra ethanol extract administration significantly increases TSP concentration in the mice given red okra extract compared to SN.

### 3.6. Histopathological Examination

**Figure 1:**
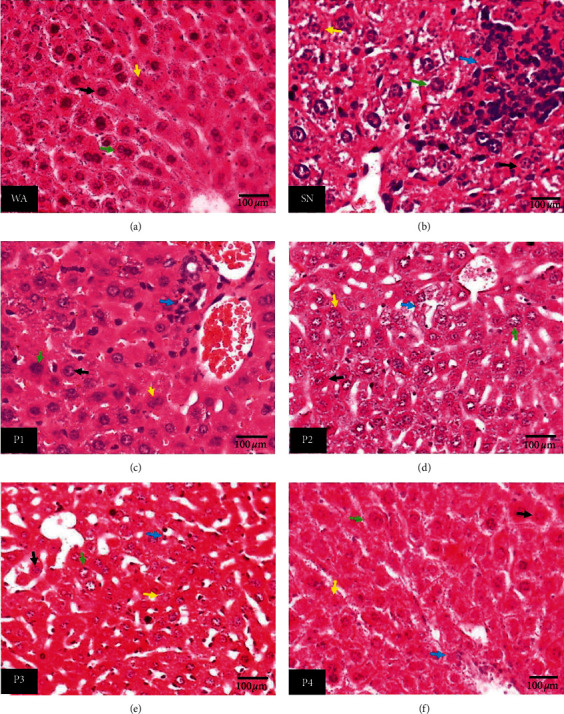
Histopathological view of liver sections. (a) Mice were given water alone. (b) Mice were given NaNO_2_ alone. (c–f) 25, 50, 75, and 100 mg/kg BW red okra pods extract and 50 mg/kg BW NaNO_2_, respectively, using hematoxylin and eosin stain technique (×400). Green arrow: swollen cell, yellow arrow: necrotic cell, black arrow: normal cell, and blue arrow: inflammation. (a) WA. (b) SN. (c) P1. (d) P2. (e) P3. (f) P4.

The SN's histological examinations showed severe inflammation (pathological score = 3, Table 3) around the portal vein, bile duct, and around the endothelia wall of the blood capillary. In this group, many hepatocytes had undergone necrosis and swelling (Figure 1, SN). The P1 group showed severe inflammation (pathological score = 3, Table 3) around the portal vein and bile duct and inflammation at the parenchyma hepatocytes accompanied by necrosis. Furthermore, the tissue of the P1 showed swollen cells (Figure 1, P1). The P2 group showed moderate inflammation (pathological score = 2, Table 3) around the portal and the parenchyma hepatocytes. The tissues displayed necrotic and swollen cells (Figure 1, P2). The P3 group showed an improved histological structure compared to P2, P1, and SN. The tissue of P3 showed mild inflammation (pathological score = 1, Table 3) at the parenchyma hepatocytes accompanied by necrosis. The cells displayed a normal structure with few necrotic cells and swollen cells (Figure 1 P3). The P4 group showed mild parenchymal inflammation infiltration (pathological score = 1, Table 3) and few necrotic and swollen cells. Generally, the P4 group showed the tissue's normal architecture and the hepatocytes (Figure 1, P4) as the WA (Figure 1, WA). Although some liver microscopic changes were available in the SN, P1, P2, P3, and P4, the severity of the effects decreased depending on the dose of ethanol extract from red okra pods administered to the mice.

**Figure 2:**
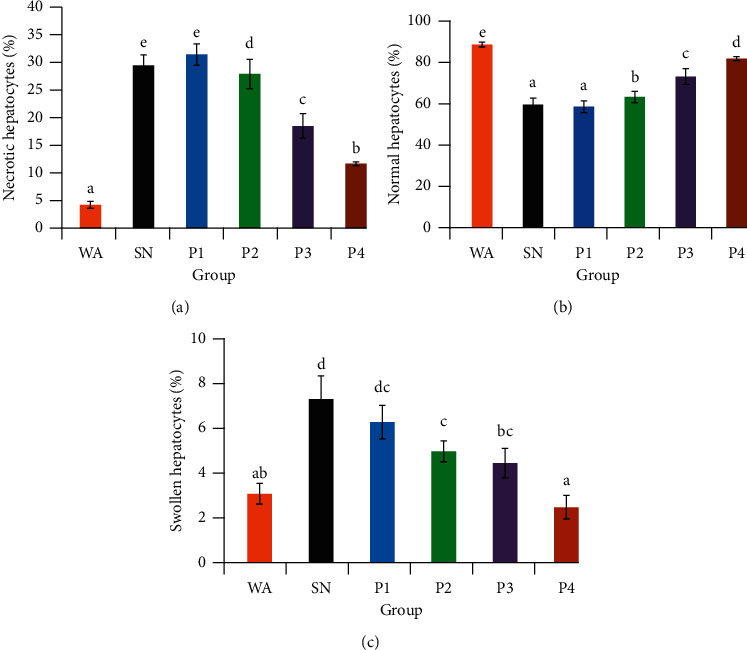
The effect of ethanol extract from red okra pods on the percentage of necrotic (a), normal (b), and swollen (c) hepatocytes. WA: mice were given distilled water alone, SN: mice were given NaNO_2_ only. P1, P2, P3, and P4: 25, 50, 75, and 100 mg/kg BW red okra pods extract and 50 mg/kg BW NaNO_2_, respectively. Different superscript letters within each figure indicate a significant difference between the means (*p* < 0.05).

The percentage of WA's necrotic cells showed the lowest percentage of necrosis, while the SN showed the highest percentage. The percentage of necrosis was significantly increased in the SN, P1, P2, P3, and P4 compared to WA. There was no significant difference between the P1 and SN. The percentage of necrosis was significantly decreased in the P2, P3, and P4 groups than the SN and P1. The P3 and P4 groups showed a significant decrease in necrosis percentage than P2 (Figure 2(a)). The WA group displayed the highest percentage of normal cells, while the SN and P1 showed the lowest percentage. The percentage of normal hepatocytes was significantly decreased in SN, P1, P2, P3, and P4 compared to the mice who received distilled water only (WA). The administration of ethanol extract from red okra pods significantly increased the percentage of normal cells in the P2, P3, and P4 compared to the SN. Generally, all groups showed a significant difference between each other except P1 and SN (Figure 2(b)).

In summary, the administration of NaNO_2_ decreased the percentage of the normal hepatocytes; however, the administration of ethanol extract of red okra to the mice exposed to NaNO_2_ increased the percentage of the normal cell compared to the mice given NaNO_2_ alone. The administration of NaNO_2_ in SN significantly caused an increase in the hepatocytes' swelling percentage compared to the WA. The administration of red okra pods ethanolic extract in P2, P3, and P4 significantly decreased the percentage of swelling compared to SN. There was no significant difference in the percentage of cell swelling between P1, P2, and P3 as well as between P1 and SN (Figure 2(c)).

## 4. Discussion

The activities of CAT and SOD were significantly increased in mice given red okra extract. The same trend of the decline in the activity of the antioxidant enzymes after NaNO_2_ exposure was reported by Salama et al. [29] and El-Nabarawy et al. [12]. This fall in the antioxidant enzymes' activities may signify the decrease of the antioxidant activity due to the oxidative stress caused by NaNO_2_. NaNO_2_ has inhibitory effects on the SOD and CAT enzymes and other antioxidant enzymes in erythrocytes [30, 31]; hence, the levels of O_2_ and hydrogen peroxide (H_2_O_2_) are elevated and cause oxidative stress [7] that can destruct other biomolecules, such as protein, lipid, and DNA [7, 11, 30, 32]. O_2_ can irreversibly oxidize non-sulfur centers of the antioxidant enzymes during protein oxidation, leading to their inactivation [33]. Another reason for the decrease in the activities may be the direct reaction of the antioxidant enzymes' metal cofactors (copper, iron, zinc, and manganese) with NO produced from NaNO_2_ to form complexes [30]. The increase of the SOD and CAT in the liver after the administration of ethanol extract from red okra pods was also reported in the study of Saravanan et al. and Hu et al. [3, 34]. In another study, okra flavonoid content caused an increase in the activity of SOD previously jeopardized by oxidative stress in fatigue-induced male mice compared to the control [35]. It was explained to support these results that okra pods have flavonoid compounds such as anthocyanin and quercetin that act as antioxidants [21, 22, 36] which scavenge free radicals [35] by donating their electrons to the oxidants [37] and hence stop the destructive chain reaction initiated by the free radicals [20]. Also, flavonoids can promote the activity of the antioxidant enzymes by activating the enzymes [38]. The activation involves various signaling cascades where the phytochemicals from red okra induce the binding of Kelch-ECH associated protein 1 (Keap1) to the nuclear erythroid 2-related factor 2 (Nrf2), leading to its stabilization, and therefore binds to antioxidant response element (ARE). This action is essential in activating the genes of the antioxidant enzymes [39, 40]. Liao et al. [41] showed that okra extracts cause upregulation of the SOD gene (SOD_2_) in type 2 induced diabetes in mice.

Moreover, flavonoids can prevent the formation of ROS/RNS. It inhibited the enzymes involved in the production of the free radicals, such as reduced nicotinamide adenine dinucleotide phosphate (NADPH) oxidase (NOx) and microsomal monooxygenase or can directly chelate metal ions such as iron that are involved in the formation of the free radicals [42], hence preventing oxidative stress that can interrupt the activity of the antioxidant enzymes.

MDA and F2-isoprostanes are essential markers of lipid peroxidation of the polyunsaturated fatty acid (PUFA), especially the arachidonic acid of the lipid bilayer of the cell membrane [4, 33]. Both MDA and F2-isoprostanes improved in the SN. The current study indicated an increase in peroxidation of the PUFA [20] due to oxidative stress. NaNO2 causes hypoxia and the production of free radicals [31] that target the side-chain methylene carbon of PUFA to remove a hydrogen atom from it to start a destructive chain reaction [43]. The decrease of the MDA and F2-isoprostanes in the red okra-treated mice, especially those given 100 mg/kg BW indicated a decrease in lipid peroxidation. The flavonoid from red okra contains three rings A, B, and C [44]. The B ring has a 3′-4′-catechol structure with a higher affinity to metal ions [42] responsible for lipid peroxidation [44], so binding the metal ions to the flavonoids prevents the metal ions from catalyzing lipid peroxidation. The decrease of MDA and F2-isoprostanes may be the red okra pods ethanol extract's role to give their electrons to the free radicals to arrest PUFA peroxidation propagation [45].

The increase of NO in SN is comparable to the findings from other studies where administration of 0.2% NaNO_2_ for six weeks caused an increase of serum NO concentration in male albino rats [46]. The increase indicated the presence of oxidative stress and hypoxia. When there is a mismatch between the oxygen demand of the cells and the amount of oxygen available in the cell, inducible nitric oxide synthase (iNOS) is overexpressed to produce more NO after being promoted by hypoxia-inducible factor-1 (HIF*α*-1) [47, 48]. Oxidative stress causes inflammation; during inflammation, iNOS that is highly expressed in the inflammatory cells, such as macrophages, is activated to produce NO that can interact with O_2_ and O_2_ to produce ONOO, which cause DNA deamination and protein nitration, hence accelerating more inflammation [48] and damage of other biomolecules [49]. During inflammation, neutrophils can activate myeloperoxidase to convert H_2_O_2_ produced from superoxide conversion by SOD to form hypochlorous acid (HOCl), a highly toxic substance [48]. Generally, the increase of NO concentration in mice administered with NaNO_2_ alone signifies the induced production of NO from iNOS. The decrease of NO concentration in mice given various concentrations of red okra pods extract compared to SN is in harmony with the study of Luo et al. [40], where flavonoids from okra flowers caused a decrease in the concentration of NO compared to the mice model with a cerebral ischemia-reperfusion injury caused by oxidative stress in the brain. Flavonoids can inhibit the synthesis of NO by downregulating the proinflammatory cofactors of iNOS induction [50]. The formation of NO during NaNO_2_ exposure depends on oxidative stress. Therefore, the best mechanism to prevent NO formation and subsequent damaging molecules such as HOCl and ONOO is to scavenge free radicals such as O_2_ and H_2_O_2_ [48]. In the current study, the increase of NO in the liver homogenates of NaNO_2_-treated animals was associated with a fall of SOD and CAT activities, while the decrease of NO levels in red okra pods-treated mice was associated with the increase of the antioxidant enzymes (Table 1). It may have confirmed that the induction of NO production facilitates free radicals, which decrease antioxidant enzymes' activities (SOD and CAT). The decrease in NO level may have confirmed the decrease in oxidative stress due to SOD and CAT activities, which have foraged O_2_ and H_2_O_2_. NaNO_2_ significantly caused an increase in ALP activity in the SN by approximately two folds compared to WA. Compared to SN, the administration of ethanol extract of red okra significantly reduced the ALP activity in all mice given the extracts. The increase of the liver enzyme activities in NaNO_2_ mice agrees with several studies [5, 6, 11, 29]; this increase indicates liver damage caused by oxidative stress, which destroys the cellular membrane leading to the discharge of the enzymes from the liver to the blood because of the increased penetrability. NaNO_2_ causes hepatocyte destruction through oxidative stress produced due to hypoxia, nitroso compound formation, or other metal-catalyzed reactions. The oxidative stress causes many destructions in the various parts of the cell, including the main target of free radicals, the cellular membrane by lipid peroxidation to decrease the plasma membrane's integrity [5] and destabilize it [6]. When the hepatocytes' cell membrane is damaged, the contents of the liver's cells, including AST, ALT, and AST, are released into the blood hence increases their activities in the serum. In this study, the serum AST, ALT, and ALP activities generally decreased in the mice given the extract compared to the SN. These results may show that the flavonoid compounds from okra earlier described by Khomsug et al. [36], Anjani et al. [22], and Irshad et al. [21] restored the normal activities of the enzymes at the highest dose (100 mg/kg BW). The results are corroborated by the study of Alqasoumi [14] and Hu et al. [34], where the administration of various doses of ethanol and methanol extracts of okra caused a decrease in the activities of liver enzymes in carbon tetrachloride- (CCl_4_-) induced oxidative stress. The reason for the decrease in the activity of the enzymes in the group given 100 mg/kg BW NaNO_2_ with various doses of ethanol extract from red okra may be the stabilization of the liver cells membrane by flavonoids to maintain the integrity of the membrane of the liver cells and protect it from the NaNO_2_-facilitated hepatotoxicity. The TSP is an essential indicator of tracing liver diseases caused by oxidative stress. The results of a decrease of TSP in the SN compared to the WA in the current study agree with Hassan et al. [6], Aboulgasem et al. [51], and Adewale et al. [52] who reported an elevated concentration of TSP in male albino rats (*Rattus rattus*), Wistar rats, and guinea pigs, respectively, treated with NaNO_2_ compared to the normal groups. The decrease may be due to the increased deamination of amino acids in the liver [53], the formation of nitroso compounds, and the inhibition of protein synthesis due to the inhibition of oxidative phosphorylation NaNO_2_ as the result of hypoxia. Saravanan et al. [3] clarified that protein synthesis inhibition might occur through ribosomal RNA hypomethylation leading to weakening lipid and protein synthesis processes. In general, the decrease in TSP may indicate the liver's failure to protect the body from oxidative stress caused by NaNO_2_. The increase of TSP concentration in the okra-treated group compared to the SN is concomitant with Alqasoumi [14]. This increase in TSP concentration may indicate the hepatoprotective potential of ethanol extract of red okra pods from the oxidative stress induced by NaNO_2_ exposure.

Usually, necrosis is accompanied by inflammation [54]. Sodium nitrite can cause a rapid depletion of adenosine triphosphate (ATP); hence, the cell membrane loses some functional properties, allowing extracellular fluids and ions. The incursion of the fluid and solutes such as sodium ions causes cytoplasm vacuolation and degeneration, swelling of the cell and organelles, and ultimately rupture of the cell membrane and, therefore, causes cell death by necrosis [55–57]. During necrosis, the contents of the cells (damage-associated molecular patterns (DAMPs)) such as histones are released due to the ruptured cell membranes, hence causing nonadaptive and acquired immune responses, including the formation and buildup of extracellular neutrophils in the tissue [58, 59]. The release of histones induces several inflammatory response cascades, such as caspase release-1 that causes cell swelling, rupture of the cell membrane, and other intracellular proinflammatory substances the accelerate liver injury. Generally, the Kupffer cells promote proinflammatory cells, monocytes, and neutrophils to the liver tissue [58].

Also, the inflammation in the liver tissue may be caused by hypoxia. NaNO_2_ causes a mismatch between the supply and demand of oxygen, causing a surge in ROS production that mediates inflammation [58, 60]. During hypoxiation, HIF-1*α* is overexpressed and translocated to the nucleus to bind with the HIF-1*β* and its cofactor p300/CBP. Overexpression of HIF-1*α* enhances the inflammatory T helper type 1 (Th1) and 17 (Th17); hence, the amount of interferon-gamma (IFN-*γ*) and interleukin-17 increases. Also, HIF-1*α* was found to potentiate the effect of interleukin 1*β* and tumor necrosis factor-alpha (TNF-*α*). The HIF aims to make the cell more glycolytic and produce ATP efficiently, hence producing lactate that enhances inflammation by boosting interleukin-6 and interleukin-23 from macrophage and succinate activates the macrophages [57]. The accumulation of macrophage around the portal area and in the parenchymal hepatocytes is associated with cells' mechanism to eliminate toxic materials to preserve the normal metabolic activities [60].

The administration of the NaNO_2_, together with doses of ethanol extract of red okra pods, showed some improvement from the damage caused by NaNO_2_. The P4 group showed the best improvement from the cellular damage and inflammation compared to the other okra-treated mice. These results are in harmony with the biochemical observations, which showed improvement of the antioxidant system of the liver and liver functions in red okra-treated animals. These findings from the microscopic examination of the hepatic tissue agree with those of Wahyuningsih et al. [20], where the administration of methanol extract from green okra caused a decrease in the percentage of necrotic cells in the kidney in male BALB/c mice exposed to lead acetate. In another study, Alqasoumi [14] found a decreased inflammation and necrosis in the liver tissues of rats administered with CCl_4_ and okra extract compared to the group given CCl_4_ only. Moreover, our current findings are supported by Al-Rasheed et al. [59], who reported that flavonoids could chelate cellular iron ions to inhibit the ubiquitination of HIF-1*α*.

## 5. Conclusion

This study shows that the administration of NaNO_2_ to mice causes damage to the antioxidant system and the liver. The administration of red okra extract has proven to possess protective potential on the antioxidant system and liver by increasing SOD, CAT, and MDA and reducing F2-isoprostanes and NO. Red okra extract succeeded in reducing the biochemical parameters of liver damage (aspartate aminotransferase, alkaline phosphatase, and alanine aminotransferase), reducing the number of necrotic liver cells and reducing swelling and hepatocytes to normal. Therefore, it can be concluded that ethanol extract from red okra has antioxidant and hepatoprotective activity against liver injury induced by sodium nitrite. Hence, we recommend the red okra pods as affordable medicines and a natural antioxidant source to protect the liver against NaNO_2_ toxicity.

## Figures and Tables

**Table 1:** Effect of ethanol extract of red okra pods on SOD, CAT, MDA, F2-isoprostanes, and NO in the liver homogenates.

Treatments	Means ± SE				
	SOD (U/mL)	CAT (U/mL)	MDA (*μ*M)	F2-isoprostanes (*μ*M)	NO (*μ*M)
WA	0.52^d^ ± 0.05	9.46^d^ ± 0.49	24.55^a^ ± 1.08	4.73^a^ ± 0.80	41.06^d^ ± 3.60
SN	0.32^a^ ± 0.02	6.08^a^ ± 0.05	86.79^c^ ± 1.88	6.49^c^ ± 0.38	58.62^e^ ± 3.38
P1	0.37^b^ ± 0.03	5.49^a^ ± 1.02	84.26^c^ ± 5.61	5.65^b^ ± 0.17	26.16^a^ ± 2.21
P2	0.42^c^ ± 0.02	6.83^b^ ± 0.43	85.61^c^ ± 3.74	6.45^c^ ±0.18	44.37^d^ ± 4.13
P3	0.46^c^ ± 0.04	7.69^c^ ± 0.65	85.02^c^ ± 2.60	6.28^c^ ± 0.30	39.99^c^ ± 0.56
P4	0.47^c^ ± 0.04	7.29^c^ ± 0.19	47.71^b^ ± 2.61	5.37^b^ ± 0.51	31.36^b^ ± 4.01

^a,b,c,d,e^Different superscript letters within each column indicate a significant difference between the means (*p* < 0.05). SOD: superoxide dismutase, CAT: catalase, MDA: malondialdehyde, NO: nitric oxide, WA: mice were given water alone, SN: mice were given NaNO_2_ alone. P1, P2, P3, and P4: 25, 50, 75, and 100 mg/kg BW red okra pods extract and 50 mg/kg BW NaNO_2_, respectively.

**Table 2:** Effect of ethanol extract of red okra pods on ALT, AST, ALP, and TSP in the serum.

Treatments	Means ± SE			
	ALT (U/L)	AST (U/L)	ALP (U/L)	TSP (mg/mL)
WA	6.61^a^ ± 1.68	7.94^a^ ± 1.67	34.07^a^ ± 8.75	60.91^d^ ± 1.91
SN	20.21^c^ ± 3.46	13.24^b^ ± 4.01	71.29^b^ ± 6.94	36.23^a^ ± 1.08
P1	18.76^c^ ± 2.43	13.23^b^ ± 1.67	60.36^b^ ± 6.08	38.97^a^ ± 1.27
P2	10.54^b^ ± 0.87	11.69^b^ ± 2.57	58.77^b^ ± 6.27	45.16^b^ ± 2.12
P3	11.76^b^ ± 3.23	9.71^a^ ± 1.60	45.40^a^ ± 7.36	44.99^b^ ± 2.34
P4	9.27^ab^ ± 1.45	8.60^a^ ± 1.39	38.14^a^ ± 6.23	50.17^c^ ± 0.96

^a,b,c,d^Different superscript letters within each column indicate a significant difference between the means (*p* < 0.05). ALT: alanine transaminase, AST: aspartate transaminase, ALP: alkaline phosphatase, TSP: total serum protein. WA: mice were given water alone, and SN: mice were given sodium nitrite alone. P1, P2, P3, and P4: 25, 50, 75, and 100 mg/kg BW red okra pods extract and 50 mg/kg BW NaNO_2_, respectively.

**Table 3:** Pathological score of the inflammation in the liver tissue.

Treatments	WA	SN	P1	P2	P3	P4
Pathological scores	0^a^	3	3	2^bc^	1^c^	1^c^

^a,b,c^Different superscript letters within each row indicate a significant difference between the means (*p* < 0.05). WA: mice were given water alone, SN: mice were given NaNO_2_ alone. P1, P2, P3, and P4: 25, 50, 75, and 100 mg/kg BW red okra pods extract and 50 mg/kg BW NaNO_2_, respectively.

## Data Availability

All crucial data used to support the findings herein are included within this article.
